# Diagnostic and Communication Challenges in Cystic Fibrosis Newborn Screening

**DOI:** 10.3390/life13081646

**Published:** 2023-07-28

**Authors:** Joan Kathleen DeCelie-Germana, Lynn Bonitz, Elinor Langfelder-Schwind, Catherine Kier, Barry Lawrence Diener, Maria Berdella

**Affiliations:** 1 Cohen Children’s Medical Center, Division of Pediatric Pulmonary and Cystic Fibrosis, Zucker School of Medicine at Hofstra/Northwell, New York, NY 11040, USA; lbonitz@northwell.edu; 2 The Cystic Fibrosis Center, Department of Pulmonary Medicine, Lenox Hill Hospital, Northwell Health, New York, NY 10075, USA; eschwind@northwell.edu (E.L.-S.); mberdella@northwell.edu (M.B.); 3 Department of Pediatrics, Renaissance School of Medicine at Stony Brook, Stony Brook, New York, NY 11794, USA; catherine.kier@stonybrookmedicine.edu (C.K.); barry.diener@stonybrookmedicine.edu (B.L.D.)

**Keywords:** cystic fibrosis, newborn screen, IRT, ethnic diversity, CF diagnostic challenges

## Abstract

As of December 2009, cystic fibrosis (CF) newborn screening (NBS) is performed in all 50 US states and the District of Columbia. Widespread implementation of CF newborn screening (CFNBS) in the US and internationally has brought about new and varied challenges. Immunoreactive trypsinogen (IRT) remains the first, albeit imperfect, biomarker used universally in the screening process. Advances in genetic testing have provided an opportunity for newborn screening programs to add CFTR sequencing tiers to their algorithms. This in turn will enable earlier identification of babies with CF and improve longer-term outcomes through prompt treatment and intervention. CFTR sequencing has led to the ability to identify infants with CF from diverse ethnic and racial backgrounds more equitably while also identifying an increasing proportion of infants with inconclusive diagnoses. Using the evolution of the New York State CF newborn screening program as a guide, this review outlines the basic steps in a universal CF newborn screening program, considers how to reduce bias, highlights challenges, offers guidance to address these challenges and provides recommendations for future consideration.

## 1. Introduction

Newborn screening for cystic fibrosis (CF) has been available in all 50 US states and the District of Columbia since December 2009 [1]. CF newborn screening (NBS) programs refer screen-positive infants to CF care centers for diagnostic sweat chloride testing and evaluation of symptoms to promote the timely diagnosis of CF, enabling early access to clinical intervention and improving outcomes [2]. CFNBS programs are most successful when experienced CF care teams work in partnership with their state’s laboratory to establish, evaluate and improve upon their CF newborn screening algorithm in accordance with the unique needs and challenges of their specific programs [1]. Using the New York State CFNBS program as our guide, we review the basic steps in all CF newborn screening programs and consider how to confront the challenges, including structural bias, that impede improved performance of screening protocols. We also discuss and provide recommendations for future areas of study.

## 2. Overview of IRT (Immunoreactive Trypsinogen)

In 1979, a breakthrough occurred in the early diagnosis of CF. It was noted that elevated levels of immunoreactive trypsinogen (IRT), a pancreatic enzyme precursor, could be measured using dried blood spots and were elevated in infants born with CF [3]. Despite lacking sensitivity and specificity as markers for CF, IRT is universally used as the initial test for CF in newborn screening programs [1]; the shortcoming of this imperfect biomarker presents both limitations and areas for improvement. Many factors can affect IRT measurements including birthweight, fetal distress, delayed testing, mishandling of specimens, storage and even seasonality [4]. The optimal IRT cut-off level for a positive result is controversial; an IRT level cut-off that is too high may miss cases and an IRT level cut-off that is too low can result in high rates of false-positive results (16) which can lead to unnecessary testing and undue stress for parents of children who test positive. Many states have adopted a floating IRT level cut-off that fluctuates based upon daily or weekly percentiles rather than a fixed IRT value [4] and some programs, typically those which routinely collected a second NBS sample from all infants, used a repeat IRT specimen as a second screening tier (IRT/IRT) [5]. Colorado was the first state in the US to undertake a CF newborn screening program in 1982 using an IRT/IRT algorithm [4]. Although IRT values can be misleading depending on the assay used by state programs, the floating IRT value cut-off has been associated with improved sensitivity and decreased missed cases of CF [6].

## 3. Brief Review of the History of Newborn Screening including Genetic Variant Panels

Given the limitations of IRT alone, the Wisconsin NBS program leveraged the discovery of the cystic fibrosis transmembrane conductance regulator (CFTR) gene, and modified its CF NBS algorithm in 1994, becoming the first US state to utilize a two-tier program that combined IRT measurements with DNA (deoxyribonucleic acid) testing for the most common CFTR genetic variant, F508del [7]. With improved sensitivity and specificity resulting from the addition of a DNA tier, the Wisconsin program reported marked success through improved identification of at-risk infants and confirmed nutritional benefits as a primary positive outcome measure [8]. The documented benefits of earlier diagnosis leading to initiation of pancreatic enzyme replacement therapy provided a clear rationale for universal CF screening of newborns and added urgency to the need for early diagnosis of those with classic CF [9]. Additional data suggest potential for improved survival in screened infants, with long-term epidemiological studies underway [10].

Other states followed Wisconsin’s lead by redesigning their CFNBS programs. Massachusetts expanded the panel of CFTR variants included in two-tier testing, with resultant gains in sensitivity and the identification of more putative CF carriers [11]. California introduced a tailored panel combined with CFTR exon sequencing (IRT/DNA/SEQ), further improving sensitivity, particularly for non-white infants less likely to be identified by the standard panel [12]. Today, all 50 US states use an IRT/DNA or IRT/DNA/SEQ algorithm. In the California model, infants with a single CFTR variant after sequencing (likely carriers) were not considered screen-positive and were not referred for CF diagnostic evaluation. Additionally, California’s results show that a considerable proportion of infants with two CFTR variants were screen-positive with negative sweat chloride test results [13]. In IRT/DNA algorithms, infants with an intermediate sweat chloride test result or, occasionally, infants with the R117H variant fall into a category of screen-positive with an inconclusive diagnosis. The utilization of genetic sequencing in CF newborn screening led to increased numbers of asymptomatic, screen-positive infants with inconclusive diagnostic test results, which highlighted a need for further guidance about how best to counsel and monitor them. Table 1 contains a summary of the three main types of CFNBS algorithms.

## 4. Quality Assurance

Universal CFNBS was instituted in all US states in 2009, but states continued to vary in the extent of CF DNA analysis, with some states screening only for the single most common CFTR variant, F508del, and others adopting a more extensive CF DNA variant panel. The infeasibility of a single standard was acknowledged by the European CF Society National Screening Working Group (ECFS) who reported that variations in culture and demographics, as well as regional and country-specific health economics, made a standard newborn screening protocol unworkable [14].

CF NBS programs have continued to examine ways of improving outcomes, such as decreasing the number of missed cases [15] and optimizing the communication between laboratories, specialty care centers (SCC), primary care providers (PCPs) and families about abnormal CF NBS results [16]. Another issue identified was the risk of missing newborns with meconium ileus, who may have low IRT levels due to intestinal blockage but have a high likelihood of having CF [17]. Kharrazi, et al. surveyed state newborn screening programs in the US with data from 29 states. They looked at factors associated with missed cases of cystic fibrosis due to IRT levels falling below the program’s cut-off. False-negative cases had a median IRT of 39 ng/mL (range 10–100 ng/mL). Factors associated with a high false-negative result were black race, programs with high IRT cut-off, fixed IRT cut-offs and genotypes without two known CF-causing variants and meconium ileus. Factors associated with a lower false-negative IRT were older age at specimen collection, Saturday birth, hotter season on newborn dried blood collection, laboratory closure of ≥3 days, preterm birth and formula feeding newborns [4]. Babies with CF born with meconium ileus are readily diagnosed due to symptoms. 

Counterbalancing concerns regarding missed cases is the goal of minimizing false-positive cases, which increases the medical burden to the health care system and emotional burden to families [18]. 

Diagnostic confirmation following a positive CF NBS result is typically performed by a CF specialty care center and includes a diagnostic sweat test according to the Clinical and Laboratory Standards Institute [19] and Cystic Fibrosis Foundation (CFF) guidelines. Sweat chloride results ≥ 60 mmol/L are considered diagnostic, 30–59 mmol/L are considered intermediate results and <30 mmol/L are negative [20]. Accuracy of sweat chloride testing is enhanced using a lab with specialized experience that takes part in regular quality monitoring and maintains a close partnership with the Cystic Fibrosis Center at their institution, as defined in the CF Foundation’s accreditation process (CFF.org). For example, sweat samples that are insufficient for analysis, or Quantity Not Sufficient (QNS), are a source of emotional distress to families awaiting diagnostic resolution [21] and may delay initiation of treatment for infants with CF. Minimizing QNS rates to <10% in newborns haw been challenging [22], particularly for premature infants and infants with low birthweight [23]. A sweat test can be performed as early as 48 h of age [24]; however, QNS rates were found to be higher in infants younger than 10 days old and premature infants [25]. The CFF recommends sweat test to be performed on an infant with a positive newborn screen or positive prenatal genetic testing by 4 weeks of age and not before 48 h old. In our shared experience, misperceptions of needing to wait until an infant is 6 or even 10 lbs. can lead to delayed diagnosis and treatment of babies with pancreatic insufficiency. Input from the CF care team regarding when to sweat test a particular baby might assist in a reduction in QNS rates. The size of the baby’s arms must accommodate a macroduct electrode to ensure adequate contact with the skin. Evaluation first by the care center may provide guidance as to when to perform the test. The decision as to when is variable depending on the size of the baby. 

## 5. The New York State CFNBS Experience

**Figure 1:**
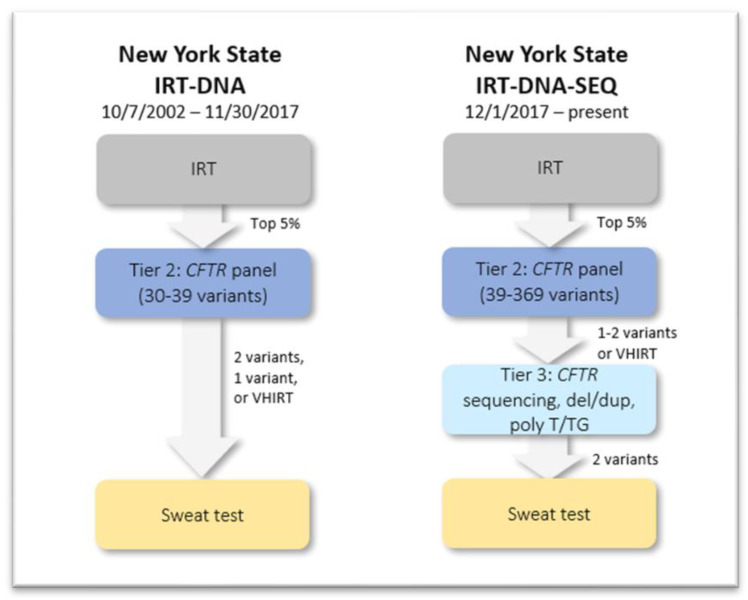
Evolution of the New York State cystic fibrosis newborn screening algorithm from inception until present. Courtesy of Denise Kay, NYSDOH (New York State Department of Health).

The New York State (NYS) CFNBS program began using an IRT/DNA algorithm in October 2002, assessing specimens from infants in the top 5% of IRT levels with an expanded CFTR panel (see Figure 1). A consortium consisting of NBS personnel from the Department of Health, CF center directors, center nurse coordinators and genetic counselors reviewed the algorithm regularly to improve sensitivity and specificity in the state’s ethnically diverse population [26]. Although data showed poor predictive value for the group of infants who presented with very high IRT levels (VHIRT) (top 0.1% for the day) and no recognized CFTR variants from the expanded CFTRpanel, who comprised the largest category of CFNBS referrals in NYS at the time, the NYS program accepted a high level of false positives to avoid missing infants with CF in this diverse population. Since the standard 39-variant CFTR panel was more likely to miss CF-causing DNA variants in infants from minoritized groups, particularly those reported as Hispanic, the NYS consortium reached consensus that the NBS program should continue to refer infants in the VHIRT category [27], but eventually raised the cut-off from 0.2% to 0.1%, leading to a large decrease in referrals without missing pancreatic-insufficient infants with CF [26].

To address these issues, the NYS CFNBS program changed its algorithm in 2017, adding a third tier utilizing next-generation sequencing (NGS) for any infant with one CFTR variant on the second-tier screening or with VHIRT and no CFTR variants on second-tier screening [28] (Figure 1). This innovation was made possible by expanded technical ability and reduced costs to the NBS program. By 2017, NGS technology made it possible to perform rapid, extensive CFTR testing in the state-run NBS lab. Eventually, the 39-variant second tier was expanded to be a targeted second-tier CFTR panel consisting of all CF-causing variants in the CFTR2 database [29]. In addition, the new testing platform was updatable as new pathogenic variants in CFTR were reported and confirmed as CF-causing. 

The IRT/DNA/SEQ algorithm enables identification of infants with CF with rare CFTR variants, resulting in fewer missed cases, especially among underrepresented populations, who are likely to have less well-defined CFTR variants [30]. Additionally, in NY, we continue to note that many cases of meconium ileus fall below the floating IRT cut-off, reported to be as high as 18% in an Italian study [31]. Absolute rates of false-negative results for this population are not available at the time of publication. With the financial, programmatic and operational support of the New York State Department of Health, including the dedication of their Newborn Screening Director and staff and the commitment of all NY (New York) CF centers, the NYS algorithm has provided an approach to CFNBS that reduces delay in diagnosis and addresses disparities by more readily identifying rare variants within the diverse population served by the NYS NBS program [26]. 

## 6. Identification of CF Carriers

Improved detection of infants with CF has also identified more carriers who are not predicted to have CF [11]. Although these infants are not expected to require any medical intervention or monitoring for CF, carrier identification has its own set of implications. How to address genetic counseling needs for this population becomes important for the infants’ parents and other relatives seeking to have children. This provides opportunity to offer genetic counseling to these individuals. It has potential implications to the infants’ future reproductive planning, with the caveat of adding the possibility for increased lifetime health risks for CFTR-related conditions [32,33]. While these are not typically considered goals of newborn screening [34], identification of carriers has been postulated as both a benefit and liability of IRT/DNA algorithms [35]. 

### 6.1. “Diagnosing” Asymptomatic Newborns

The possibility of an inconclusive diagnosis exists within the framework of all CF newborn screening algorithms but is heightened when CFTR sequencing results are reported [36]. Diagnostic ambiguity after CF newborn screening may stem from an intermediate sweat chloride test result in an infant with at least two CF-causing CFTR variants, or identification of one or more variants of uncertain significance (VUS) and/or varying clinical consequence (VCC). Note that VCC is a term used to describe CFTR variants [37] but is not currently a formal variant classification as defined by the American College of Medical Genetics [38]. This diagnostic scenario is termed CFTR-Related Metabolic Syndrome (CRMS) in the US [39].

The European CF Society’s Neonatal Screening Working Group further characterized the CRMS designation as Cystic Fibrosis Screen Positive Inconclusive Diagnosis (CFSPID). An international panel of experts proposed the term CFSPID in 2014 as a more descriptive and clinically correct designation for this large group of asymptomatic and genetically diverse patients [40]. The European Working Group spearheaded the development of guidelines for infants with inconclusive CF diagnosis after newborn screening. Representatives from 30 countries, including the US, reached consensus on proposed recommendations using Delphi methodology. Follow-up recommendations included evaluation by a physician from a CF specialty center or with CF expertise, repeated sweat chloride testing at 6–12 months, close contact with a local pediatrician, clear guidance to the family with need for follow-up given the increased risk for a future diagnosis of CF or CFTR-related conditions, and annual re-evaluation of the specific CFTR variants identified in each patient [41]. 

Additional international efforts published in 2017 provided a global harmonization of the CRMS and CFSPID designations [42]. The acronym CRMS continues to be used predominantly in the US while the more descriptive CFSPID is commonly used outside the US. The term CRMS implies illness when these variants may never be clinically significant [14,43], whereas CFSPID implies a need for monitoring and follow-up to clarify the infant’s proper designation over time, including the possibility of remaining healthy. The use of the term CRMS addresses the need for a unique diagnosis category for use by payors in the US. CRMS has a unique ICD-10 (E88.9) billing code on the platform used for reimbursement for US clinicians and hospitals. This diagnostic code enables providers to follow babies with inconclusive diagnosis after a positive CF NBS result, monitor them according to clinical management guidelines, and receive reimbursement for provision of these services. 

Currently, such monitoring includes repeat sweat chloride testing, expanded genetic testing for the infant and parents, and other clinical tests such as fecal elastase, sputum cultures, chest X-rays and fat-soluble vitamin levels. Additional testing is decided based on the cystic fibrosis physician’s clinical judgment [20]. Assessments of CFTR function via nasal potential difference and intestinal current measurement have demonstrated utility in reclassifying infants from CFSPID to CF and identifying those at risk for developing CFTR-related conditions [44] but are not widely used in children in the US. 

### 6.2. *CFTR* Variant Interpretation Challenges

Incorporation of a CFTR sequencing tier into CF NBS programs has increased identification of variants not well characterized as CF-causing. Proper identification of the clinical significance of a novel or rare CFTR variant is still an evolving area of study in medicine, with guidelines for variant classification set by the American College of Medical Genetics and Genomics (ACMG) setting the standard [38]. In the context of a VUS or VCC, clinicians must interpret complex and uncertain genetic findings for the family of an asymptomatic infant. 

Several public data repositories contain information about CFTR variants. The CFTR Mutation Database housed at the Hospital for Sick Kids in Toronto, Canada [45], maintains a database of the >2000 CFTR variants but reports limited clinical information. An invaluable repository of genetic and phenotypic information from individuals with a CF diagnosis is the CFTR2 database [29]. The French CF Association also maintains a searchable database that includes a wider phenotype than CFTR2, including CFTR-related conditions [46]; other sources of information can be found by searching for the variants in genomic databases such as Clinvar or gnomAd as well as PubMed or even web-based searches. Reviewing published reports about rare CFTR variants may be helpful when counseling families about the need for continued follow-up, recognizing that there may be variable phenotypes with the same or similar genotypes. Genetic counselors, who are trained to synthesize and communicate complex genetic information, are inconsistently associated with CF care centers for CF newborn screening follow-up [47].

### 6.3. Health Disparities in CF Newborn Screening

In our NY City experience, being in an urban area, we have found a multitude of challenges within our community, including the follow: 1. changes of address from the time of birth; 2. changes in surnames that are not readily noted on a newborn screening Guthrie card; 3. communication access to operational phones and computer networks for emails; 4. economic burden of lack of paid time off (PTO) in parental employment; 5. transportation difficulties to the CF center; 6. primary care setting within a clinic of multiple providers without ownership of the patient; and 7. lack of readily available interpretive phone service. These issues were heightened by the COVID-19 pandemic. It is important to create a plan as part of pandemic and disaster preparedness as such challenges are predicted to recur in the future.

The lack of inclusion of rare CFTR variants in newborn screening algorithms increases the likelihood that infants from minority groups (black/African American, Hispanic and other race) are missed disproportionately in the newborn period [48], resulting in a delay in diagnosis [49] that leads to worsened pulmonary, nutritional and cognitive outcomes [50]. This may further manifest in lower life expectancy as compared to those with earlier diagnosis and access to therapies. Individuals who have CF due to common CFTR variants such as F508del are more likely to be white and eligible for highly effective modulator therapies [34]. Children on CFTR modulators have an improved quality of life with reduced need for standard CF medications and respiratory treatments [51].

## 7. Longer-Term Implications for Access to Treatment of Rare, Modulator-Ineligible Variants

Newborn screening algorithms that include NGS identify individuals with less common CFTR variants. As ivacaftor is approved for infants as young as one month of age [52] and elexacaftor/tezacaftor/ivacaftor is approved for those at least 2 years of age, it is imperative to identify newborns who will benefit from early treatment. Individuals from minoritized groups are more likely to have rare variants, some of whom could respond to modulators for early treatment [46]. If a rare variant is not approved for treatment, one potential way to address the disparity is through theratyping [53]. The process of theratyping assesses whether a particular variant may be responsive to CFTR modulators by testing in vitro at designated research laboratories. Clinicians can use theratyping results to advocate for patients to trial modulators off-label or as an n-of-1 study. Positive outcomes including stabilization or improvement of pulmonary function and reduction in pulmonary exacerbations for patients with rare CFTR variants have been reported [54].

## 8. Implications for Primary Care Practitioners

Inconclusive CFNBS results lead to a significant challenge for primary care practitioners (PCPs), who are often the first to receive notification of abnormal screens. PCPs are integral to the process of referring babies with an abnormal CFNBS result for diagnostic resolution and particularly for monitoring babies with CFSPID, who may not exhibit any symptoms in the pediatric or adolescent age groups. The importance of providing education to PCPs about recognizing symptoms that prompt rapid referral to CF centers cannot be overstated. As CFNBS algorithms change, providing clear information to PCPs is important to avoid unnecessary referrals and sweat tests. Letters of communication with PCPs regarding positive newborn screen results should use language that is concise and leaves less interpretive errors. The work of Sontag, et al. highlights the importance of strong alliance and communication between primary care physicians (PCPs), State NBS programs and local CF care and research centers [1].

## 9. Psychosocial Considerations

As the newborn screening landscape continues to evolve with advanced genetic technologies and changing interpretations of CFTR variants, consideration of the psychological impact to parents and families, potential fiscal impacts and ethical concerns is important. In the summer of 2021, the UK National Screening Committee commissioned a task force to elicit input from a sample of the public who had no previous knowledge of cystic fibrosis, prior to the adoption of next-generation sequencing (NGS) in CFNBS. The project aimed to determine whether making a prompt diagnosis of cystic fibrosis was important. Even when faced with an increased number of missed newborns with a CF diagnosis, the public felt that avoiding the uncertainty of a CFSPID diagnosis was just as important a goal of a CFNBS program [53]. These findings reflect a central conflict in deciding to expand CFTR evaluation as part of the CFNBS as use of a limited CF genetic panel may not address the ethnic diversity within populations [27], while wider genomic screening can mitigate these disparities and shortcomings [51,52]. However, implementation of NGS will result in more families receiving an uncertain diagnosis. The waiting period for sweat testing and its results can be very distressing for parents. Cognitive understanding of carrier status and cystic fibrosis, the physician’s approach to the parents and adjustment to the new infant may affect the level of distress [55].

The communication of specialty care teams with PCPs is critical in recommending the right language to convey newborn screen results and sweat testing results. This is particularly challenging when the diagnostic status of the infant remains inconclusive after sweat testing, due to the presence of one or more VUS or VCC. Multi-center and long-term studies of the psychosocial impact of a positive CFNBS result, both with or without the additional complexity of an inconclusive diagnosis, have identified parental worry and anxiety as well as concerns about increased perceptions of child vulnerability [18]) associated with a positive CFNBS result. However, in an early study comparing parents of babies with CF, parents of babies with inconclusive diagnoses, and healthy controls, Perobelli et al. noted that parents of asymptomatic babies with inconclusive diagnoses viewed their babies as healthy, as did parents of healthy controls. Parents of inconclusive diagnoses and healthy controls experienced less distress than parents of babies with positive diagnosis of CF [56]. 

Tluczek, et al. published a systematic review in 2022 synthesizing findings from 92 evidence-based, peer-reviewed research reports about psychosocial issues associated with newborn screening. The expansion of genetic variant detection will affect minoritized groups at a larger scale, requiring added support in communicating positive results, whether it pertains a true CF diagnosis or CRMS/CFSPID [57]. The use of interpretative services may be needed and challenging. Access to certified personnel in medical interpretation is not always easily available but the use of tablets in CF clinics may offer a reasonable alternative to clinicians in a face-to-face encounter as wall-mounted phones lack the opportunity for this. The increased number of ethnicities identified in our NY experience highlights the importance of having the proper time, knowledge, sensitivity and communication skills to work with varying populations. Parents and families may require additional encounters to convey information and provide extra emotional support regarding NBS results. Parents are often sleep-deprived and are concerned about their baby when they come in to meet the CF team and receive the newborn screening results. Parents may not always be ready to receive the amount and type of information that we as the CF team may want to provide. Clinicians must remain cognizant of the varying needs of parents, the risk and/or presence of postpartum depression and the impact on other children in the family as these factors can impair their capacities to absorb and assimilate new information [57]. This process of CF newborn screening, diagnosis and communication of difficult and, at times, inconclusive results is a lengthy process including multiple team members, effective communication and ongoing follow-up. 

## 10. Clarifying the Diagnostic Status: CFTR Phasing

Another diagnostic challenge follows identification of a baby with one or more rare CFTR variants, as parental phasing is needed to determine the inheritance pattern of the variants. The process of CFTR phasing is critical in differentiating a likely heterozygote, with all variants inherited in cis from the same parent, from an infant with variants on both copies of CFTR who needs ongoing monitoring for CFSPID/CRMS. A consequence of the phasing process might be the identification of parents who have either two variants or complex alleles themselves and who are asymptomatic or mildly symptomatic [58]. These results require additional time for explanation to the parents. It is appropriate that referral to an adult cystic fibrosis center for further evaluation and genetic counselling be offered. Of note, CFTR phasing also has utility for parents and families of infants with a clear CF diagnosis. Understanding the segregation of the variants can inform carrier screening options, genetic counseling and test selection. 

## 11. Implications of Prenatal Modulator Exposure

Identification of CF in pregnancy raises added communication needs for interfacing with CFNBS programs [59]. A recently published case report [60] suggests that babies with CF born after prolonged prenatal exposure to CFTR modulators may have IRT values below the cut-off, leading to false-negative CFNBS results [61]. A second case report describes a heterozygous carrier mother who received ETI (elexacaftor/tezacaftor/ivacaftor) after her fetus was diagnosed with cystic fibrosis and bowel obstruction was noted on ultrasound [62]. Resolution of the bowel obstruction suggests that prenatal exposure to CFTR modulators may alter the clinical outcome of the disease. Ongoing communication between prenatal care providers, CF centers and NBS programs is crucial to avoid false-negative CF newborn screening results that cause delay in appropriate care and follow-up. 

## 12. Conclusions

Since the institution of universal newborn screening in 2009, the evolution of diagnostic methods has improved diagnosis and care of CF newborns in the US and internationally. The addition of CFTR sequencing leads to more accurate diagnosis and many positive changes for children with CF by improving nutritional status and instituting earlier interventions at specialized care centers that ultimately increase life expectancy. Although advances in genetic testing methods allow for more expansive CFTR variant identification in newborn screening algorithms, the use of NGS leads to an unintended but expected increase in the diagnosis of CRMS/CFSPID cases. Examining long-term benefits and potential psychosocial consequences for this population is an area for continued research. 

New York State has one of the most ethnically diverse populations in the US. The experience of the NYS CF NBS program highlighted challenges in early CF diagnosis, the expansion of the genetic variant panel, incorporation of CFTR sequencing, an increased number of inconclusive diagnoses and apparent disparities as it underwent major changes and evolved into the current IRT/DNA/SEQ algorithm. These challenges prompted quality improvement efforts and ongoing partnership between the state and specialty care centers. The continued reevaluation of our algorithm, ongoing collaboration with our state newborn screening program and commitment to the inclusion of all ethnicities may provide guidance in efforts to revise the CF newborn screening programs in other communities at the local level. At the state level, cystic fibrosis center teams are encouraged to meet with members of Department of Health newborn screening programs to continue to improve detection rates, decrease the number of false-positive tests and improve outcomes in their individual programs. In partnership with NBS laboratories, PCPs and CF care providers can aid in advocating for a more equitable CFNBS program. The implementation of third-tier sequencing, which has been successful in California and New York CFNBS models, is one approach. With highly effective modulator therapies now available in infancy, there is a new imperative for early identification and fair treatment of all babies with CF.

Our goal and our hope are that medical professionals, government bodies and other decision makers work toward a more uniform approach to the early diagnosis of cystic fibrosis newborns, promoting equity, reducing harm and avoiding delayed diagnosis in all babies with cystic fibrosis.

## Figures and Tables

**Table 1:** CF newborn screening algorithms.

IRT/IRT	Initial measurement of immunoreactive trypsinogen (IRT); elevated IRT results are repeated on a separately collected specimen on a different day.
IRT/DNA	Single elevated IRT is followed by DNA analysis from the same blood spot specimen to detect the most common CF variant(s).
IRT/IRT/DNA	Second elevated IRT is followed by DNA analysis from the same blood spot specimen to detect the most common CF variant(s).
IRT/DNA/NGS	Single elevated IRT is followed by DNA analysis from the same blood spot specimen to detect the most common CF variant(s). If 2 CF variants are not identified, the specimen is reflexed to next-generation sequencing of CFTR.

Description of the methodologies utilized for newborn screening in the United States. Next-generation sequencing technology in New York State includes analysis of the CFTR coding region, intron/exon boundaries, specific intron variants and large exon deletions/duplications (del/dup) using bioinformatics. Minimum sequencing coverage depth was 10×. Standard or real-time PCR was used to confirm large del/dup. All possible del/dup and variants deep in introns or the promoter regions are not detected by these methods.

## Data Availability

No new data was created.
